# Quinine in Continued Fever

**Published:** 1853-11

**Authors:** 


*Quinine in Continued Fever.*—The advocacy of the exhibition of this drug in the continued fevers, by Dr. Fenner, of New Orleans, has given rise to some spirited controversy in that city. Dr. Scruggs is the principal opponent of its use.

We are sorry to notice that the controversy has degenerated into a somewhat personal attack, and that a number of side issues have been raised. Dr. Fenner insists that the question should be brought to the test of experiment, and submits that an innovation so startling and variant from our preconceived notions, should be judged rather by the *experimentum crucis*, than by theoretical argument. He does not assert the inefficiency of the usual treatment, but declares his belief that quinine goes beyond the mere sustaining of the vital powers, and is abortive in its effects, tending to cut short the disease, and to hasten a favorable termination.

The authorized treatment of the continued fevers—that one upon which the best medical minds are now united in opinion—looks not to the abortion of the disease, but to such a support of the vital forces as may best enable the system to endure the effects of the blood poison which constitutes the disease, and thus to retain its integrity during the process of elimination.

To this end are given ammonia, brandy, nutritious diet, opium, small doses of quinine, and some of the special stimulants, as ol. terebinth. By these the depressing effect of the morbific poison upon the vital forces is lessened, the nervous system sustained, and enabled to wear out the process of elimination.

This process of elimination is said to be self-limited, and is neither to be hastened or assisted. Here is the point of theoretical variance between Dr. Fenner and his opponents. Dr. F. concedes to quinine a power of exalting the nervous system above the influence of the poison, of carrying the vital forces to such a point of energy and activity, that the process of elimination is hastened, and the disease cut short.

Or perhaps it acts as a counter-poison. It is now conclusively settled that the virus communicated by the rattlesnake, (in every sense of the word a zymotic poison,) may be overcome by the poison of alcohol. A drunken person is bitten by a rattlesnake with no serious results. Or a sober person being bitten, and the system coming deeply under the influence of the poison, this influence is overcome, and the progress of the poison checked, by the exhibition of large quantities of alcholic drinks.

This is theory — or rather it is a theoretical application of well-known facts. We do not know that quinine acts in any such manner to cut short a fever. The point first to be settled is, whether it does cut it short. If it does not, we shall be illy repaid for our theorizing — if it does, we can find plenty of time hereafter to account for its mode of action.

And here we pause in our argument, to insert some cases reported by Dr. Fenner, in the New Orleans Medical Journal. We have not space for the insertion of Dr. Fenner's argument preceding his reports of cases, and only room for a portion of the cases themselves, but in omitting it, though our readers will lose the perusal of an able paper, we shall still be following the suggestion of Dr. Fenner, who says:

" It is useless to discuss its theory any further. Let us bring it at once to the test of experience."

Case 1. M. H., a robust Irish girl, aged 20, entered the hospital on the 3d of April; was taken sick the day previous. Says she was attacked with chilliness, soon followed by hot fever, with severe pains in the head, back and limbs. These symptoms continued when she was admitted. The house student prescribed a dose of castor-oil.

*April* 4—I saw her for the first time. She has high fever, face flushed, skin hot and dry, pulse 122, tongue heavily coated, brown and dry in the center, edges whitish, tip red, abdomen full and tympanitic; bowels freely purged by the oil.

*Treatment.* ℞. S. quinine, ℈j.

Tinct. opii, ʒss.

Aqua minth.

Pip. ʒss. M. S.

Take all at once.

To have also liquor ammoniæ acetat. ℥iv. A tablespoonful every two hours.

*Evening*—Condition much the same. To have sulphate quinine, ʒss; blue mass, grs. viii; pulv. opii, grs. ii. M. Take at one dose.

*April* 5—Looks better, rested well, skin moist but hot, pulse reduced to 104, tongue moist, abdomen still tympanitic, but not tender, bowels open, slight roaring, but no pain in head.

*Treatment.*—Continue the acetate of ammon. during the morning. Evening—To have sulph. quinine, ʒss; pulv. opii, grs. ii. M. Take at one dose.

*April* 6—Says she feels very well; looks better, face still flushed, skin moist and cooler, sweated profusely during the night, pulse increased to 120, tongue still brown in center but moist, bowels open, less tympanites.

℞ S. quinine, ʒss.

Pulv. opii, gr. i.

Take at once. M.

*Evening*—Found her bathed in perspiration, pulse reduced to 100, tongue moist and cleaning, no headache, slight roaring in head, bowels open, slight tenderness and tympanites.

℞ S. quinine, grs. xxv.
Pulv. opii, grs. ii.
Take at once. M.

*April* 7—Much better, rested well, sweated freely, skin cool and moist, pulse 90, full and soft, less tympanites and no tenderness, tongue more natural. To have 10 grains quinine. Continue acetat. ammonia.

*April* 8—Greatly improved, fever completely broken, pulse 65, no tympanites, tongue moist and white. Complains more of the roaring in the head than when she took the large doses of quinine. To have lemonade and no medicine. From this time she continued to improve, had no return of fever, and was discharged on the 16th.—*Notes by Mr. McKnight.*

*Remarks.*—Here we see a severe case of continued fever, (*typhus* or *typhoid*,) in which the abortive treatment was commenced in good time, and the fever cut short in *four days;* in which time she took 145 grains of quinine, 7 grains of opium, and ʒss tinct. opii. It will be observed that there was less *quininism* whilst taking the large doses than the small, a fact well known to the physicians of New Orleans.

Case II. Catharine Murphy, aged 18, Irish emigrant, came on the ship "Samuel Lawrence," with case No. 1; felt unwell from the time she landed here, was dejected in spirits, but did not say when she was attacked with fever. Entered the hospital on the 4th of April.

*Symptoms.*—Pains in the head, back, and limbs, skin hot, face flushed, bowels loose, thirst, pulse 120, tongue thickly coated, edges red, stomach irritable, abdomen tympanitic but not tender on pressure.

*Treatment.*—To have a dose of castor-oil, and after it operates, the following:

℞ Sulph. quinine, ʒss.
Mass. hydrarg., grs. viii.
Sulph. morphia, gr. ss.
Take all at once. M. Make pills.

She threw up the oil but retained the pills.

*Second day, April* 5—Feels no better, rested rather badly, pains continue, face still flushed, skin moist, pulse as before, bowels open, tongue rather dry, brown and thick, little sordes on the teeth, impatient and restless.

*Treatment.*—To have aqua ammonia acetatis during the day; at night to have the following:

℞ S. quinine, ʒss.

Pulv. opii, grs. ii.

Take at one dose. M.

*Third day*—Feels a little better; skin moist and cooler, less headache, tongue more moist, some tympanites, pulse 115, stomach still irritable.

*Treatment.*—Same as yesterday, acetate of ammonia through the day; quinine, ʒss. and opium, grs. ii. at night.

*Fourth day*—"Feels no better," face and eyes somewhat injected, expression listless, sweating pretty freely, pulse 110, tongue coated, thick and red on the edges, tympanites, *sudamina* over the abdomen and chest, has some cough, debility not great.

*Treatment.*—Blisters to epigastrium and nucha.

℞ Sulph. quinine, ℈i.
Sulph. morph, gr. ss.
Take at one dose. M.

*Fifth day*—Feels better, slept well, still drowsy, bathed in perspiration, less pain in head and limbs, pulse 74.

*Treatment.*—As the fever is now subdued, stop the quinine, but continue the acetate of ammonia, lemonade and light soup.

*Sixth day*—Feels a great deal better, but still has slight uneasiness in head and limbs, pulse 85, skin cool and sweating, tongue moist and cleaning, no tympanites, bowels open but not loose, countenance more expressive and calm. Wants to sit up, and says, "she would be entirely well now if she could get plenty to eat."

*Treatment.*—Liniment for limbs, lemonade and soup.

*Seventh day*—Rested well, pains entirely gone, has a good appetite, fairly convalescent. She continued to improve till the 14th April, when she was discharged from the hospital. After remaining out a few days, she had a relapse and returned to the hospital; but the fever yielded readily to treatment, and the girl was again discharged cured.—*Notes by Mr. Taney.*

*Remarks.*—Here again was a severe and obstinate case, that would, in all probability, have run on for twenty or thirty days under ordinary treatment; but by the plan pursued it was cut short on the sixth day of treatment. She took 210 grains of quinine, 1 of sulph. morphia, and 4 of opium. She seems never to have complained of quininism at all.

Case 3 was similar to these. The fever had progressed three days before treatment; but yielded to quinine on the fourth day.

CASE IV. Ann Sawyer, aged 35, has lived in the city two years; was admitted on the 26th March; been sick seven days. Ordered by the ward student ol. ricini, ℥jss.

*March* 27—First seen by Dr. R.

*Symptoms.*—Hot and dry skin, small and frequent pulse, tongue heavily coated, bowels freely opened by the oil.

*Treatment.* ℞ Mass hydrarg., grs. x.

Sulph. quinine, ℈j.

Sulph. morph., gr. ss.

At one dose. M.

*Evening*—To have a scruple of quinine and half a drachm of tinct. opii, in a little mint water. (This is the haustus quiniæ of the house.)

*March* 28—Found her a great deal better, pulse 95, skin cool and moist, feels entirely easy, bowels open.

*Treatment.* ℞ Sulph. quinine, ℈ji.
Pulv. opii, grs. ii. M. Make two powders.
Take one morning and evening. Lemonade, half diet.

29—Still better, pulse 90, tongue moist and cleaning, skin cool and moist, has slight uneasiness in bowels, which are rather loose.

*Treatment.* ℞ S. quinine, grs. xv.
Pulv. opii, grs. jss. M. In two powders.
One morning and night. Mistura cretæ for diarrhœa.

30—Cool and quiet, bowels easy, pulse 80, tongue nearly clean. She continued to improve from this date, and was discharged in a few days.

In the male wards of the hospital, (my own,) I treated a number of emigrants who came over on the snip "Samuel Lawrence.". They were taken sick at different periods after their arrival: some within a few days, others after twenty or thirty days. Some had gone to work in the city; others on the plantations. They all presented the same type of fever, but entered the hospital in different stages: some recent, others advanced. All the cases which had not advanced beyond the third or fourth day, were promptly cut short by the quinine and opium treatment. I will report two cases that came together, which will show both the efficacy of the treatment and the nature of the disease. These men had come over in the same ship, and had gone to work together ditching, on a plantation above the city. One was treated with quinine, the other without it.—*Reported by Mr. McKnight.*

Case 5 was fatal. No quinine was given. Dr. Fenner remarked "that he was too far advanced for the abortive treatment, for he did not claim to cut the fever short after the third or fourth day of illness, that he only gave the quinine before any organic lesion had taken place."

Case VI. Thomas Fletcher, an Irishman, came over in the same vessel with the other, and was working in company with him, aged 40 years; entered the hospital April 24th, has been sick four days; taken with a slight chill followed by a slight fever with aching in his bones for several days. skin moist, pulse 100 per minute, tongue dry, slightly coated, bowels regular, abdomen tympanitic, tenderness over epigastrium.

*Treatment.*—To be cupped freely over the stomach, afterward the following:

℞ Sulph. quinine, grs. xxv.
Mass. hydr., grs. x.
Sulph. morphiæ, gr. ss. M. Make pills.

Take at one dose.

*April* 25—Appearance about the same, skin hot but moist, pulse 100, tongue moist and coated white, abdomen tympanitic, no tenderness over epigastrium, bowels open.

*Treatment.*—Nothing ordered this morning. At 5 o'clock, P. M., the student found him in a kind of stupor, skin cool, tongue moist, pulse weak but frequent, 112 per minute; and gave him some carbonate of ammonia.

*April* 26—Much better, no pain, moist tongue, pulse 100.

*Treatment.*—Ol. ricini, ℥i; enema purg. to be taken if oil does not operate.

*April* 27—Still better, seems to be improving.

*Treatment.* ℞ Quinine solution, ℥vi.
Take a tablespoonful three or four times a day.

*April* 28—Decidedly convalescent. Continue the small doses of quinine. He continued to take the solution of quinine, and was discharged on the 2d of May, perfectly well.

*Remarks.*—It will be perceived that this patient took but one large dose of quinine, after which the excitement became so low as to demand a stimulant. His convalescence was rapid and he had no relapse, but it can hardly be doubted that if the case had gone without treatment as long as the previous did, it would have been difficult to cure. The two cases having been similarly exposed and attacked, were most probably the same disease.

Cases 7 and 8 do not vary from this latter in any essential facts. In case 7, though the fever had progressed ten days, there seemed to be no organic lesion. It convalesced on the third day. Case 8 commenced treatment earlier, and convalesced on the third day. S. B. H.

